# Influence of Carbon Nanotube Clustering on Mechanical and Electrical Properties of Cement Pastes

**DOI:** 10.3390/ma9040220

**Published:** 2016-03-23

**Authors:** Sung-Hwan Jang, Shiho Kawashima, Huiming Yin

**Affiliations:** 1 Department of Civil Engineering and Engineering Mechanics, Columbia University in the City of New York, 610 Seely W. Mudd 500 West 120th Street, New York, NY 10027, USA; sunghwaj@andrew.cmu.edu (S.-H.J.); yin@civil.columbia.edu (H.-M.Y.); 2 Robotics Institute, School of Computer Science, Carnegie Mellon University, 5000 Forbes Avenue, Pittsburgh, PA 15213, USA

**Keywords:** carbon nanotube, clustering, mechanical property, electrical conductivity

## Abstract

Given the continued challenge of dispersion, for practical purposes, it is of interest to evaluate the impact of multi-walled carbon nanotubes (MWCNTs) at different states of clustering on the eventual performance properties of cement paste. This study evaluated the clustering of MWCNTs and the resultant effect on the mechanical and electrical properties when incorporated into cement paste. Cement pastes containing different concentrations of MWCNTs (up to 0.5% by mass of cement) with/without surfactant were characterized. MWCNT clustering was assessed qualitatively in an aqueous solution through visual observation, and quantitatively in cement matrices using a scanning electron microscopy technique. Additionally, the corresponding 28-day compressive strength, tensile strength, and electrical conductivity were measured. Results showed that the use of surfactant led to a downward shift in the MWCNT clustering size distribution in the matrices of MWCNT/cement paste, indicating improved dispersion of MWCNTs. The compressive strength, tensile strength, and electrical conductivity of the composites with surfactant increased with MWCNT concentration and were higher than those without surfactant at all concentrations.

## 1. Introduction

Multi-walled carbon nanotubes (MWCNTs) have superior mechanical, electrical, and thermal properties compared to other carbon-based materials [1,2,3]. Because of their excellent material properties and high aspect ratio, many applications have emerged in electric devices and multi-functional materials [4,5]. During the past decade, there has been much research progress in the field of civil engineering on the utilization of MWCNTs to enhance mechanical [4,6], thermal [7,8], and electrical properties [9,10,11] for the development of multi-functional cement-based composites. For example, Chung [12,13] has studied the electrical properties of cementitious-based composite with conductive materials for many applications, such as strain sensors, electromagnetic shielding, and thermal interface. In addition, Baeza *et al.* [14] used conductive cement composites with carbon nanofiber as strain and damage sensors. At the same time, the importance and challenges of effective CNT dispersion have become apparent. Konsta-Gdoutos *et al.* [15,16] demonstrated that the mechanical properties of cement composites depend highly on the dispersion of the MWCNTs. They proposed an effective protocol and achieved excellent flexural strength compared with other previously-published works.

Strong van der Waals forces that arise from the high specific surface area of CNTs and polar characteristics of water make dispersion of MWCNTs in water difficult [17]. To overcome these problems, a number of processing methods have been employed, including the use of surfactants [18,19], functionalized CNTs [20,21,22], silica fume [11,23], and ultrasonication [24]. In most studies, the evaluation of dispersion has been limited to aqueous systems, although it is reasonable to expect the dispersive state to be different in the highly alkaline environment found in cement-based systems. In fact, MWCNTs considered to be initially highly-dispersed in an aqueous form were observed to be aggregated in cement matrices using scanning electron microscopy [25]. Further, evaluation of dispersion in the cement matrix has mostly been limited to qualitative analysis. Therefore, the effective distribution of MWCNTs, once they are introduced into the cement matrix, is still unclear. Dispersion remains a prominent issue in various systems, including infrastructural material systems, where a network of well-dispersed MWCNTs is difficult to achieve. Therefore, for practical purposes, it is of interest to evaluate the impact of MWCNTs at different states of aggregation/dispersion on the eventual performance properties of cement-based materials.

The present study investigated the dispersion of MWCNTs in both aqueous and cement-based systems, and the resultant effects on mechanical and electrical properties when incorporated into cement composites for potential application as multi-functional materials. The MWCNT chosen for the study exhibits relatively low purity (~85%) to reduce cost and increase the feasibility of scaling up for use in construction. Research grade MWCNTs can be expected to cost $2300/25 g. The industrial grade MWCNTs used in this study cost $300/25 g, so approximately 10% of the cost of higher purity forms. Clustering of the MWCNTs was controlled through surfactant treatment and ultrasonication. MWCNT/cement pastes were prepared with different concentrations of MWCNTs, ranging from 0% to 0.5% by mass of cement. MWCNT clustering was assessed qualitatively in an aqueous solution through visual observation, and quantitatively in cement matrices by measuring MWCNT clustering size distribution using a scanning electron microscopy technique. The compressive strength, tensile strength, and electrical conductivity of cement paste incorporating different concentrations of MWCNTs were measured. The effect of surfactant, and subsequently degree of MWCNT clustering, on each of these properties is discussed.

## 2. Experimental Section

### 2.1. Materials

**Figure 1:**
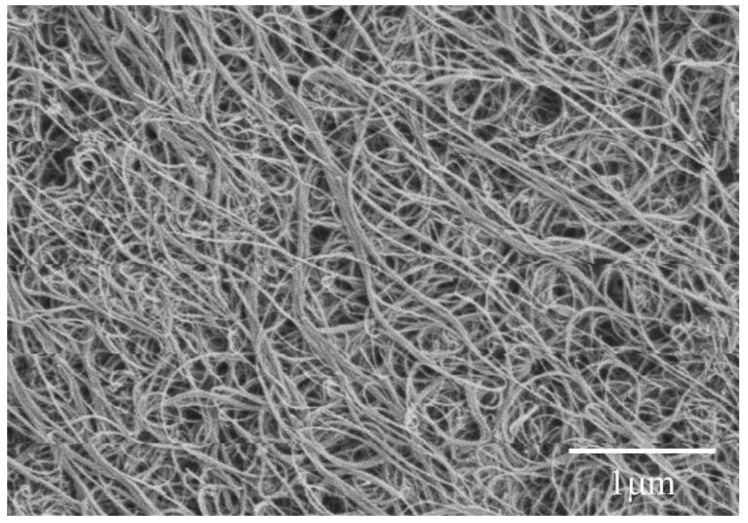
SEM morphology of as-received multi-walled carbon nanotubes (MWCNTs).

Type I Portland cement was obtained from Lafarge (Chicago, IL, USA) and the chemical composition is listed in Table 1. MWCNTs were obtained from Nanolab Inc. (Waltham, MA, USA). They were produced using chemical vapor deposition. Table 2 presents the material properties of the MWCNTs and Figure 1 shows the SEM morphology, in which agglomeration exists. The purity of the MWCNTs was approximately 85 wt % and their diameters and lengths were 10–30 nm and 5–20 μm, respectively. A polycarboxylate-based water reducing admixture surfactant (ADVA Cast 575) was obtained from Grace Corporation (Cambridge, MA, USA). The surfactant provides steric hindrance or static charge repulsion for stabilization of nanomaterials, which had been found to be effective for the dispersion of MWCNTs in water [26,27,28].

### 2.2. Sample Preparation

Different concentrations of MWCNTs (0%, 0.05%, 0.10%, 0.25%, and 0.50% by mass of cement) were considered in this study. Guided by previous studies [21,23], a relatively high range of MWCNTs was tested in order to clearly investigate their effects on electrical conductivity when incorporated into cement paste. Water-to-cement ratio was chosen to be 0.5 for the sample preparation. MWCNTs were added, as-received, to the mixing water with/without surfactant to control the MWCNT clustring, then sonicated using a horn-type ultrasonicator for 30 min. The operating frequency of the ultrasonicator was 22 kHz and operated in pulse mode, *i.e.*, cycles of 15 s on and 15 s off. The sample was submerged in an ice bath throughout the sonication process to prevent evaporation. The operating conditions of the ultrasonicator were based on the protocol developed by Konsta-Gdoutos *et al.* [15]. Then, the suspension was added to the cement and mixed in a high-shear mixer for 4 min. For all tests, the specimens were demolded after one day and air-cured at room temperature for 28 days.

### 2.3. Characterization

The clustering states of MWCNTs in both aqueous solution and cement paste were investigated using both visual observation and scanning electron microscopy (SEM) (Hitachi 4700 SEM, Tokyo, Japan), respectively. We evaluated the clustering of MWCNT in aqueous solution with different concentrations of surfactant after being left to rest for 72 h or after centrifugation. For the latter, MWCNT suspensions were subjected to centrifugation in a tabletop centrifuge at 10,000 rpm for 10 min. We also used SEM to measure the diameters of MWCNT clustering in the cement paste. SEM images were acquired randomly along the fracture surfaces of the 0.05%, 0.1% and 0.25% MWCNT/cement composites. The sampling size was 50.

**Figure 2:**
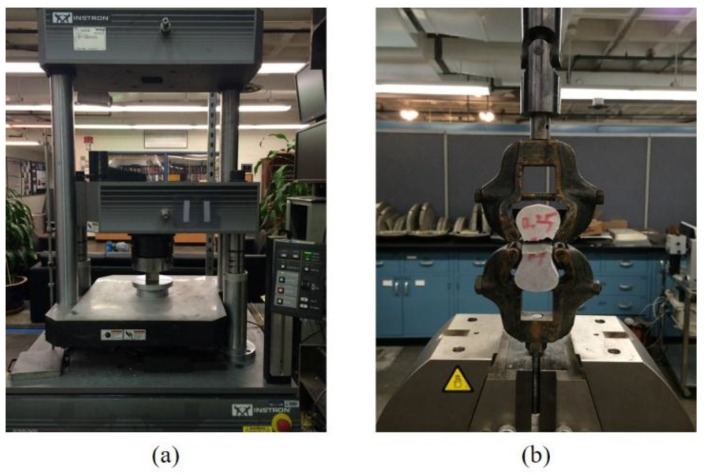
Measurement of mechanical properties: (**a**) Compressive test and (**b**) Direct tensile test.

For all mechanical and electrical characterization, three samples were tested for each mix after 28 days of curing and the average was taken to be the representative value. The compressive strengths of 50.0 mm cubic specimens were tested on an INSTRON (Norwood, MA, USA) 600DX 135 k Universal Testing Machine (Figure 2a). The tensile strength of dog-bone shaped specimens with a minimum center cross section of 30.0 mm × 20.0 mm were tested on an INSTRON 5984 34 k Universal Testing Machine at a rate of 0.1 mm/min (Figure 2b). Due to the brittle nature of cement pastes, some samples experienced fractures at the supports. The authors would like to note that those samples were discarded. Only samples fractured at the tapered section were reported and the variability, indicated by the error bars in the results, was within acceptable limits.

**Figure 3:**
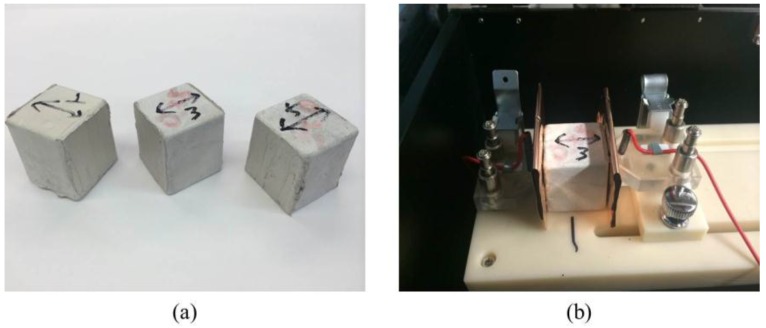
Measurement of electrical conductivity: (**a**) Silver painted samples; (**b**) set-up for volume resistance measurement.

The volume resistance of 25.5-mm cubic samples were measured at room temperature using a Fluke 8846A (Everett, WA, USA). The setup is shown in Figure 3. The electrical conductivity of each sample was measured in accordance to ASTM C1760 by using direct current (DC). In this study, the two-probe method was employed [29]. Although it contains a contact resistance between the probe and surface of the samples, it provides relatively consistent measurements compared to the four-probe method. In addition, it also provides clear cross-sectional areas of the electrodes, which is useful for calculating the electrical conductivity of the specimen. On the other hand, the four-probe method may provide higher accuracy through reduced contact resistance effects, but it requires mesh-type electrodes and the insertion depths of the electrodes may vary—as a result the cross-sectional area must be approximated. Notice that the contact resistance of this test configuration can be reduced to such a low level compared to the high resistance of the composite sample, the two-probe method is able to provide comparable accuracy plus other advantages, as mentioned. In order to minimize contact resistance, high purity silver paint was applied to both ends of the specimens, between the test probe and the electrode of the composites. The electrical conductivity of the composites can be calculated from:

(1) $$\sigma =\frac{L}{AR}$$

where R is the volume resistance of the composite, A is the area of the electrode, and L is the distance between the electrodes.

## 3. Discussion

### 3.1. CNT Clustering in Water and Cement Paste

**Figure 4:**
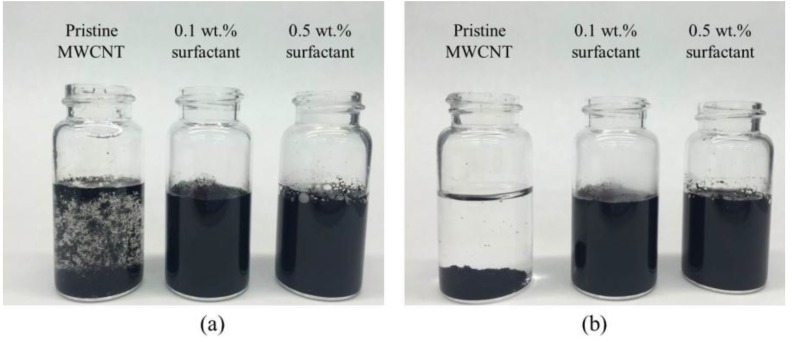
Stability of MWCNT suspension after (**a**) 5 min; (**b**) 72 h.

In this Section, 0.1% of MWCNTs by mass of cement were dispersed in water with surfactant concentrations of 0%, 0.1%, and 0.5% by mass of cement. Recall, all samples had a w/c ratio of 0.5. Figure 4a shows the dispersion of MWCNTs in aqueous solution just after sonication and Figure 4b shows the dispersion after 72 h. MWCNTs in the suspension without surfactant immediately aggregated and settled to the bottom, indicating unstable MWCNTs in pure water. However, it is apparent that MWCNTs with surfactant showed improved dispersion, where uniform suspensions were observed after 72 h and even up to one month.

**Figure 5:**
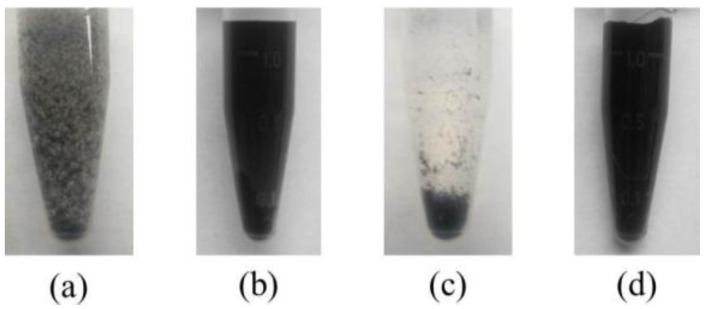
Stability of MWCNT suspension. (**a**) Untreated with surfactant before centrifugation; (**b**) treated with surfactant before centrifugation; (**c**) untreated with surfactant after centrifugation; (**d**) treated with surfactant after centrifugation.

To further test stability, MWCNT suspensions were subjected to centrifugation. The results are shown in Figure 5. It is evident that MWCNT suspensions without the surfactant exhibited aggregation (Figure 5a), where most of the MWCNTs fell to the bottom after centrifugation (Figure 5c). MWCNT suspensions with 0.1% surfactant also exhibited visible aggregation. However, MWCNT suspensions with 0.5% surfactant remained uniform after centrifugation (Figure 5d), so relatively stable. Since the surfactant helped MWCNTs to decrease surface tension, as well as overcome van der Waals interactions between MWCNTs, higher surfactant content led to improved stability. These observations indicate that the surfactant is an effective dispersant for MWCNTs in aqueous solution and 0.5% by mass of cement is a suitable dosage.

**Figure 6:**
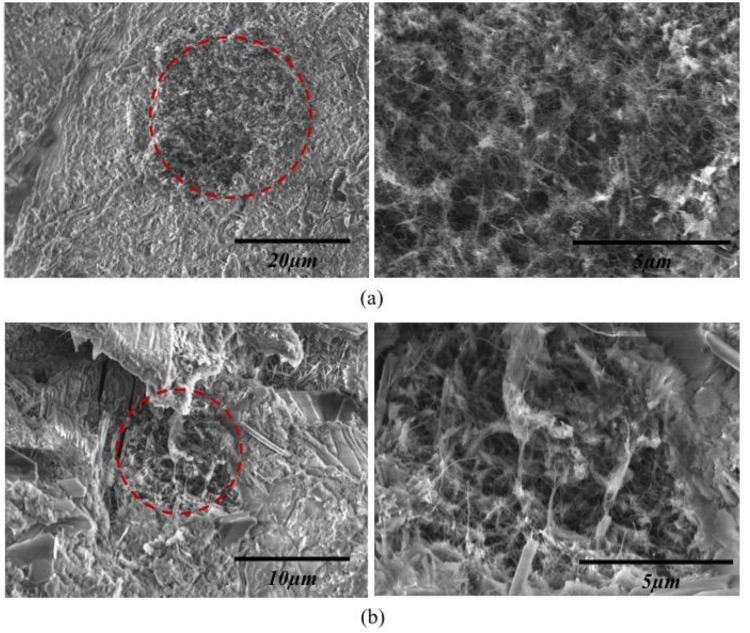
SEM images of representative MWCNT aggregates in MWCNT/cement composites. (**a**) Untreated with surfactant; (**b**) treated with surfactant.

Dispersion of MWCNTs in cement matrices is expected to be different from its dispersion in aqueous systems. Although some studies have presented effective dispersion methods for CNTs in cement-based materials, it still remains a challenging task due to high alkaline environment found in these systems. Figure 6 shows SEM images of 0.1% MWCNT/cement composites. All specimens showed some clustering of MWCNTs, regardless of the concentration of MWCNTs or use of surfactant. Previous studies have found that uniform dispersion of CNTs in cement pastes cannot be achieved, even with the use of surfactant, due to the high alkalinity present in cement-based systems [25,30]. For further investigation of MWCNT dispersion in cement matrices, the size distribution of the MWCNT clustering was evaluated using an SEM imaging technique.

**Figure 7:**
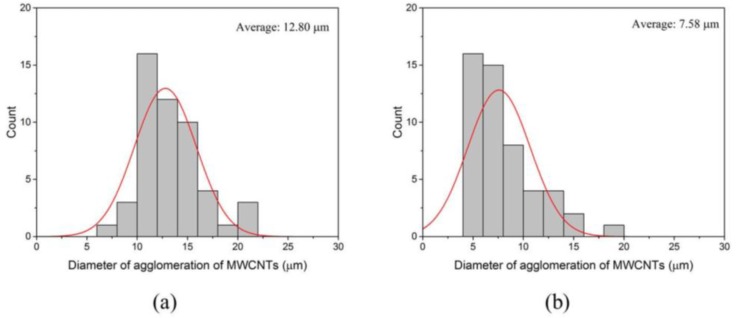
Distribution of MWCNT aggregates in 0.05%, 0.1%, and 0.25% MWCNT/cement composite. (**a**) Untreated with surfactant; (**b**) treated with surfactant.

Figure 7 shows the results for MWCNT/cement composites with and without surfactant. Larger diameters of clustering (*D*_avg_ = 12.8 μm) were dominant in MWCNT/cement composites without surfactant, whereas smaller clusterings (*D*_avg_ = 7.6 μm) were found in the MWCNT/cement composites with surfactant. The results indicate that, although MWCNT clustering still occurs with the surfactant, using surfactant is still beneficial for the dispersion of MWCNTs in cement matrices. How the dispersive states of the MWCNTs affect the mechanical and electrical properties of the composites in the preceding sections will be discussed.

### 3.2. Influence of CNT Clustering on Mechanical Properties

**Figure 8:**
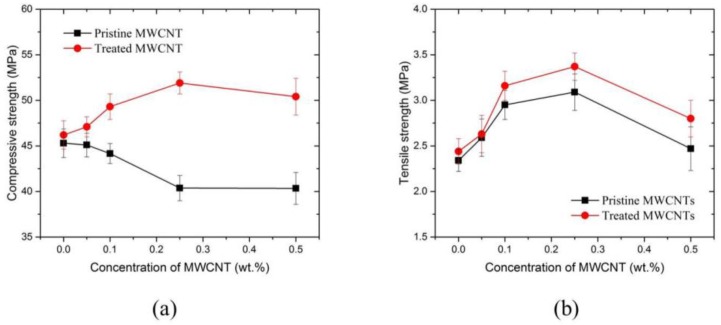
Mechanical properties of MWCNT/cement composite at 28 days. (**a**) Compressive strength; (**b**) tensile strength.

The compressive and tensile strengths of MWCNT/cement pastes at 28 days are shown in Figure 8. In this study, 0.5 wt % of surfactant was used for all samples for mechanical and electrical properties. Three samples were tested for each mix design, from which the average was taken to be the representative strength. Overall, MWCNT/cement pastes with surfactant showed greater enhancement in compressive and tensile strengths compared to the composites without surfactant. The composites with surfactant showed an increase in compressive strength with MWCNT content up to 0.25% MWCNT, reaching a 20% increase compared to the control. In contrast, composites without surfactant exhibited a decrease in compressive strength with increase in MWCNT content throughout. A similar result was found in Reference [19]—for a given dispersion protocol, using a suitable surfactant was necessary to improve mechanical properties. On the other hand, increase in tensile strength with MWCNTs was observed in both composites, with and without surfactant, as shown in Figure 8b. The highest tensile strength was achieved at 0.25% MWCNT in the composite with surfactant, with an increase of 40% with respect to the control sample. However, a significant drop in the tensile strength was observed at 0.5% due to severe agglomeration of MWCNTs, but the MWCNT/cement composite with surfactant still showed higher strength compared to the control.

**Figure 9:**
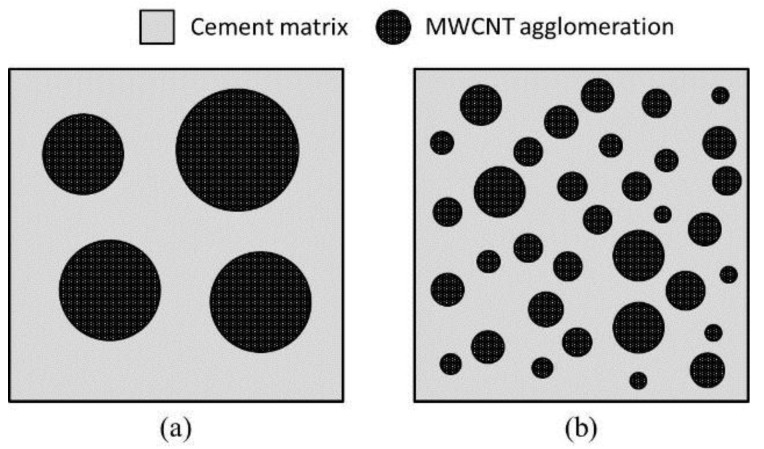
A schematic representation of MWCNT/cement composite. (**a**) Untreated with surfactant; (**b**) treated with surfactant.

There is a hypothesis that nucleation of hydration products in CNTs promotes and accelerates cement hydration [31]. As MWCNTs can act as nucleating agents for calcium silicate hydrates (CSH), which is a major component for mechanical improvement, this can at least partially explain the higher mechanical strength of the MWCNT/cement paste compared to the control sample. The reduction in compressive strength of MWCNT/cement composites without surfactant may be attributed to clustering of MWCNTs due to poor dispersion, resulting in air voids in the microstructure (Figure 9a). On the other hand, the increase in compressive strength of MWCNT/cement composites with surfactant may be attributed to improved dispersion of MWCNTs and also surface enhancement of MWCNT in the cement matrices (Figure 9b). Improved distribution of MWCNTs can improve particle packing, leading to a denser microstructure [26]. Moreover, well-dispersed MWCNTs could arrest and bridge cracks in the cement matrix to suppress crack propagation at the nanoscale [32].

### 3.3. Influence of CNT Clustering on Electrical Conductivity

**Figure 10:**
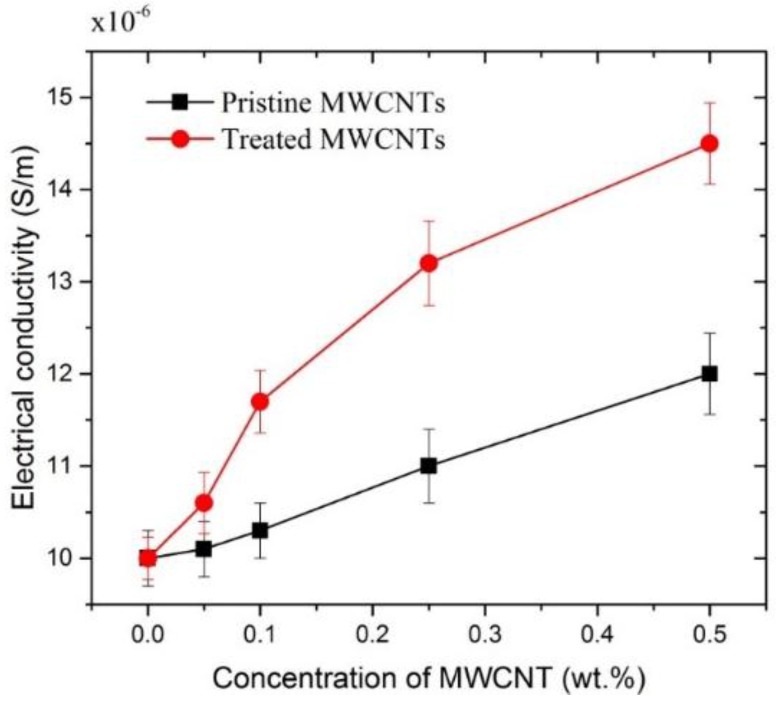
Electrical conductivity of MWCNT/cement composite at 28 days, treated and untreated with surfactant.

The electrical conductivity of MWCNT/cement composites is an important property, as change of electrical conductivity has been used to indicate the strain and fracture in sensor applications. The electrical conductivities of MWCNT/cement paste with/without surfactant are shown in Figure 10. Three samples were tested for each mix design, from which the average was taken to be the representative conductivity. The electrical conductivity of cement paste without MWCNTs was ~1.0 × 10^−5^ S/m, regardless of surfactant, indicating that the surfactant does not affect overall electrical conductivity. It can be clearly seen that the electrical conductivity of the MWCNT/cement pastes increased with increasing concentration of MWCNTs. Further, electrical conductivity of the MWCNT/cement composites with the surfactant exhibited higher electrical conductivity than those without surfactant. For instance, the electrical conductivity of 0.5% MWCNT/cement pastes with/without surfactant is about 50% and 20% higher, respectively, than the electrical conductivity of plain cement paste.

The higher electrical conductivity of MWCNT/cement paste with surfactant may be caused by the improved dispersion of MWCNTs, leading to less clustering. MWCNT is a conductive material with a relatively high electrical conductivity of 10^3^–10^5^ S/m, whereas cement hydrates in this study have a relatively low range of 10^−6^–10^−3^ S/m. This contrast supports the fact that the electron transport takes place through a MWCNT network in the cement matrix, consisting of both singly dispersed MWCNTs and clustering. Therefore, the total electrical resistance of MWCNT/cement pastes with surfactant may be much lower because MWCNTs are better distributed throughout the cement matrix and effectively creating other electron paths (Figure 9).

It is worth noting that, due to some intrinsic physical characteristics of the composite system, such as the curvature of the MWCNTs, re-clustering of MWCNTs in alkaline environment, and porosity of cement paste, the electrical conductivity of the MWCNT/cement pastes were not significantly improved compared to other MWCNT composites, polymer-based composites in particular [33,34,35]. However, compared to other nanoinclusions, such as carbon black and graphene nanoplatelets [36], MWCNTs were still found to be effective in achieving higher mechanical and electrical properties of cement-based materials at a relatively small amount. Further investigation on the rheology of MWCNT/cement composite will be needed for practical application in civil engineering.

## 4. Conclusions

We have demonstrated the influence of CNT clustering in both aqueous solution and cement matrices; and on the mechanical and electrical properties of MWCNT/cement pastes. The surfactant was found to reduce the clustering of MWCNTs in cement matrices compared to MWCNT/cement composites without surfactant, based on visual evaluation and measurement of average aggregate diameter of MWCNTs in cement matrices through SEM. The compressive and tensile strength of MWCNT/cement composites with surfactant showed improvement at up to 0.25% MWCNTs, indicating an optimal concentration for mechanical performance. Electrical conductivity of the MWCNT/cement composites with surfactant was higher than those of composites without surfactant throughout, and increased with increasing MWCNTs up to 0.5% MWCNTs. This is caused by better distribution of MWCNTs in cement matrices due to surfactant treatment, which facilitated the formation of a network of highly conductive MWCNTs and reduced the total resistance of the MWCNT/cement composites. MWCNT/cement paste shows potential to serve as a multi-functional infrastructure material due to its enhanced electrical and mechanical properties [7,37,38,39,40]. Future research will include exploring practical applications for this advanced composite system in civil engineering, e.g., strain sensing and damage sensing, with considerations of cost as well.

## Figures and Tables

**Table 1:** Chemical composite of cement.

Oxide	Composition (wt %)	Mineralogical Phase	Composite (wt %)
CaO	63.7	Tricalcium silicate (C3S)	52.4
SiO_2_	12.9	Dicacium silicate (C2S)	16.0
Fe_2_O_3_	7.9	Tricalcium aluminate (C3A)	10.2
SO_3_	5.3	Tetra-calcium aluminoferrite (C4AF)	8.6
Al_2_O_3_	4.2	Magnesite	0.3
MgO	3.5	Calcite	0.5
K_2_O	0.9	Dolomite	1.2
TiO_2_	0.3	–	–
ZnO	0.2	–	–

**Table 2:** Material properties of MWCNT.

Properties	Values
Density	2.3 g/cm^3^
Surface area	250–300 m^2^/g
Diameter	10–30 nm
Length	5–20 μm
Electrical conductivity	1 × 10^5^ S/m
Purity	≥85 wt %
